# Supplementary figures and images for: Parallel evolution of reduced cancer risk and tumor suppressor duplications in Xenarthra
Source: eLife. 2022 Dec 8;11:e82558. doi: 10.7554/eLife.82558 (PMC9810328; doi:10.7554/eLife.82558)

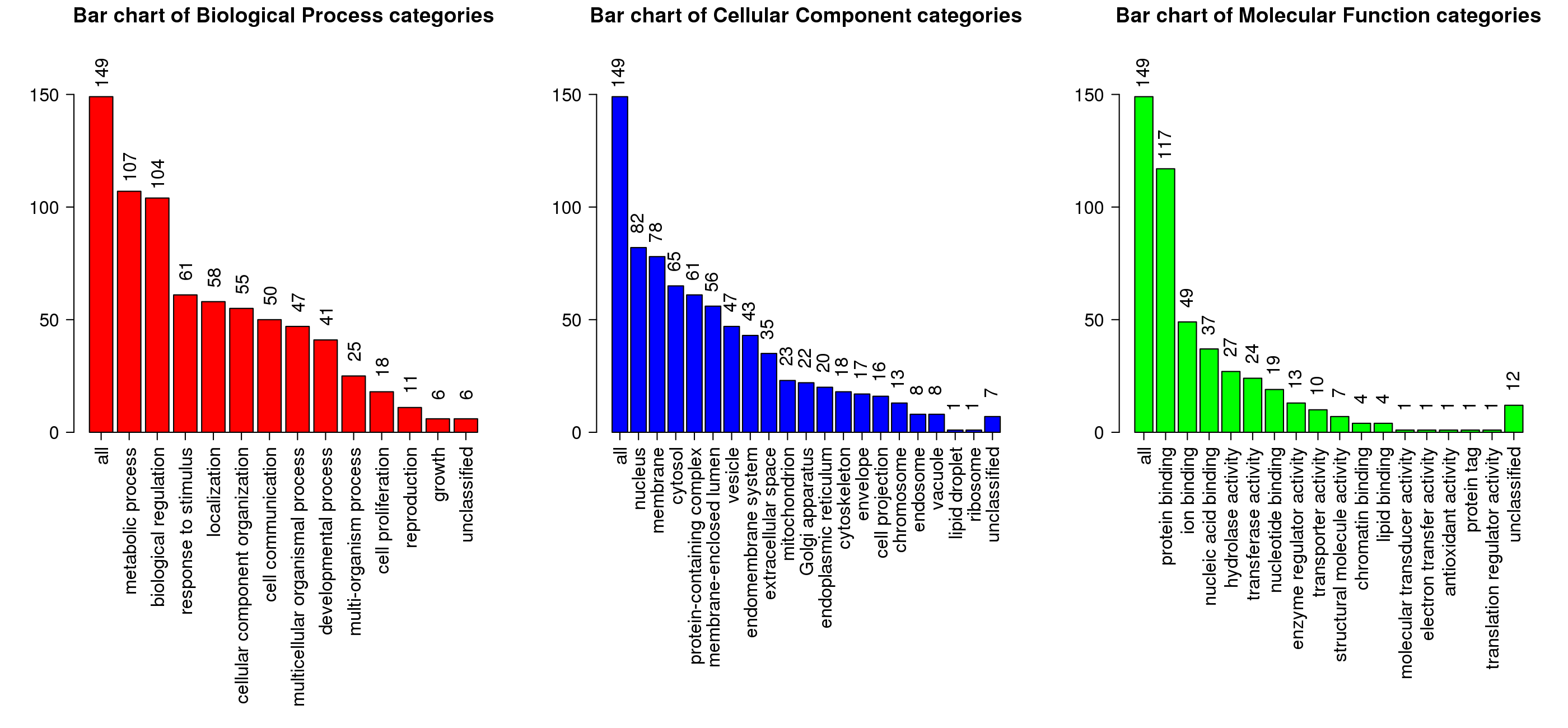

Supplement: Figure 3—source data 4. [file elife-82558-fig3-data4.zip › Figure 3 ΓÇô source data 4/goslim_summary_wg_result1657297622.png]

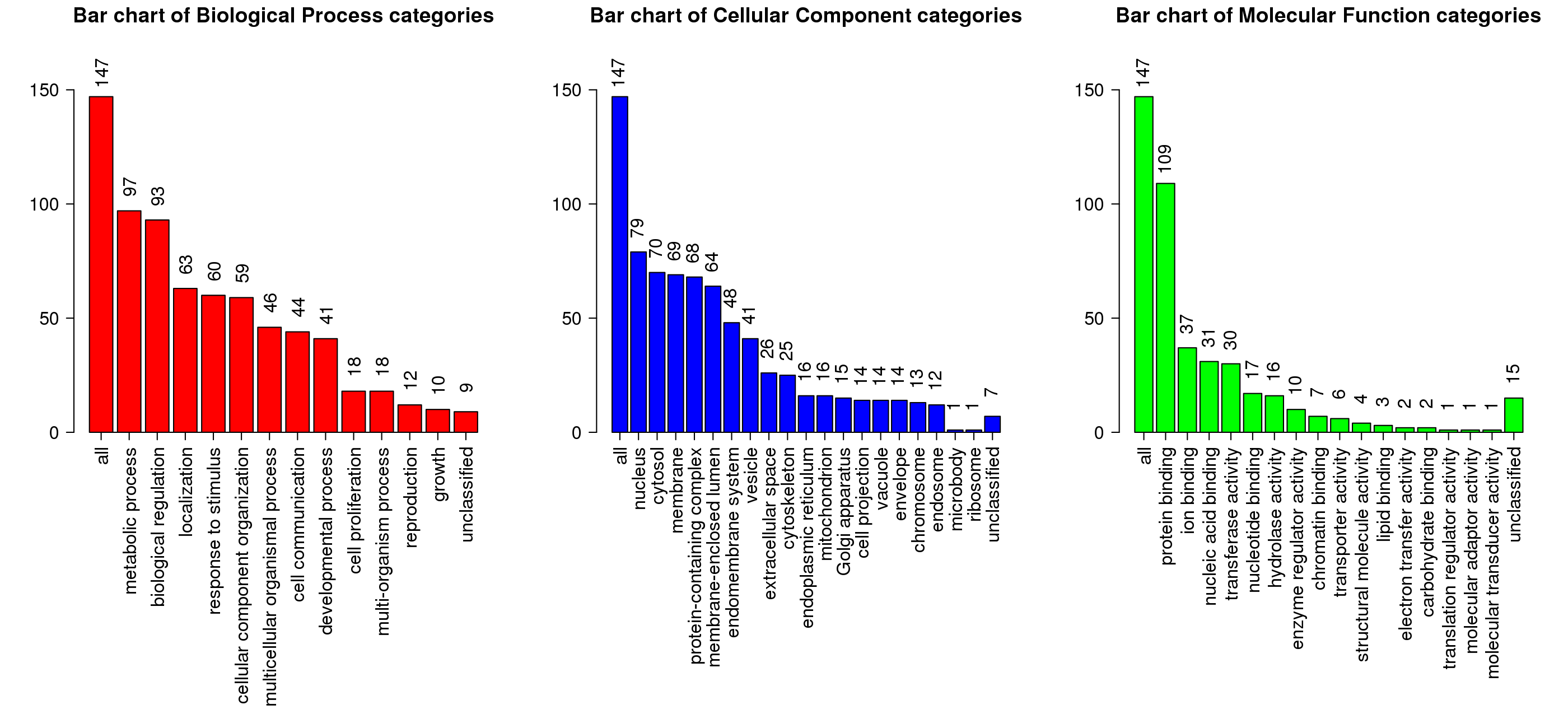

Supplement: Figure 3—source data 5. [file elife-82558-fig3-data5.zip › Figure 3 ΓÇô source data 5/goslim_summary_wg_result1657298103.png]

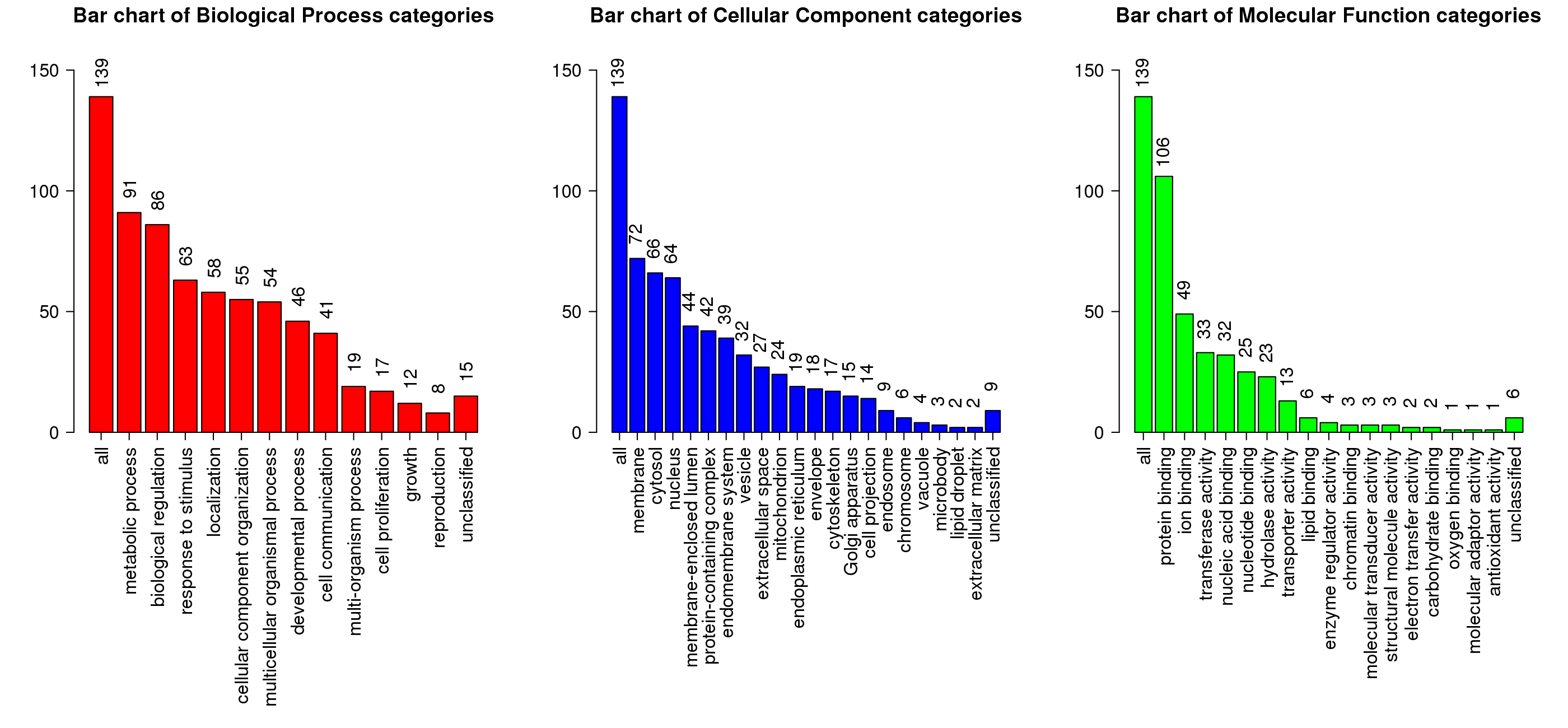

Supplement: Figure 3—source data 6. [file elife-82558-fig3-data6.zip › Figure 3 ΓÇô source data 6/goslim_summary_wg_result1657298133.png]
